# Supplementary material for: Spatiotemporal imaging and shaping of electron wave functions using novel attoclock interferometry
Source: Nat Commun. 2024 Jan 12;15:497. doi: 10.1038/s41467-024-44775-5 (PMC10786904; doi:10.1038/s41467-024-44775-5)
Supplement: Supplementary file 1 — Supplementary information [file 41467_2024_44775_MOESM1_ESM.pdf]

Supplementary Information for

Spatiotemporal imaging and shaping of electron wave functions using novel attoclock interferometry

Peipei Ge[1,2], Yankun Dou[1], Meng Han[3], Yiqi Fang[1], Yongkai Deng[1], Chengyin Wu[1], Qihuang Gong[1,4,5], Yunquan Liu[1,4,5*]

[1]State Key Laboratory for Mesoscopic Physics and Frontiers Science Center for Nano-optoelectronics, School of Physics, Peking University, Beijing 100871, China

[2]Wuhan National Laboratory for Optoelectronics and School of Physics, Huazhong University of Science and Technology, Wuhan 430074, China

[3]J. R. Macdonald Laboratory, Department of Physics, Kansas State University, Manhattan, Kansas 66506, USA

[4]Collaborative Innovation Center of Extreme Optics, Shanxi University, Taiyuan, Shanxi 030006, China

[5]Peking University Yangtze Delta Institute of Optoelectronics, Nantong 226010, Jiangsu, China

I. Saddle-points of strong-field ionization by 400 nm circular fields

We numerically solve the saddle-point equation in single 400 nm circular fields for each final momentum **p** and present the saddle-points with their real part t_r confined within [0, T_{400}] as shown in Supplementary Fig. 1. It is clear that the ionization time t_r is uniformly mapped to the emission angle. And it does not depend on the photoelectron energy. While for the imaginary part t_i, it reveals an isotropic property along different emission angles and tightly depends on the photoelectron energy. As t_i quantifies the time the electron spends under the potential barrier, the result in Supplementary Fig. 1b indicates that the low-energy electrons spend more time to pass through the barrier than the high-energy electrons.

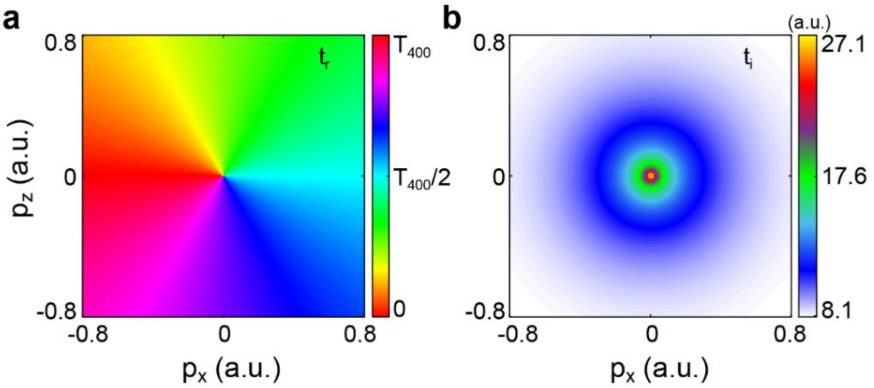

Supplementary Fig. 1| Derived momentum-resolved saddle-points t_s for strong-field ionization by 400 nm circular fields within [0, T_{400}]. a Real part t_r of the saddle-point. **b** Imaginary part t_i of the saddle-point.

II. Comparison among the experimental results, strong-field approximation (SFA) calculations and Coulomb-corrected SFA(CCSFA) calculations

In CCSFA calculation, we adopt the saddle-point approach to derive the complex ionization instants of electrons. Based on the complex ionization instants, we then calculate the amplitude, phase and ionization exit of the initial electron wave packet. Afterwards, we propagate the electron wave packet in the combined laser and Coulomb fields. The calculated two-color phase-resolved photoelectron energy spectra at the emission angles of θ=0°, 45°, 90° and 135° using CCSFA model are shown in Supplementary Fig. 2i-l. For comparison, we also present the measured results in Supplementary Fig. 2a-d together with the SFA calculated results as shown in Supplementary Fig. 2e-h. It is clearly visible that, influenced by the Coulomb potential, the yield of low-energy electrons in the energy spectra increases, indicating that the radial momentum of electron wave packet is decreased due to the Coulomb attraction. Moreover, the phase-resolved interference fringes in the cases of θ=45°and 135° as shown in Supplementary Fig. 2j and 2l slightly shift in low-energy region as compared with the SFA calculations and they show good agreement with the experimental results. This demonstrates that the discrepancy between experiment and SFA calculation as shown in Fig. 2 in the main text does arise from the Coulomb effect during the classical propagation of electrons.

Importantly, one can notice that, the interference patterns calculated by SFA model in the cases of θ=0°and 90° (Supplementary Fig. 2e and 2g) are basically consistent with that calculated by the CCSFA model (Supplementary Fig. 2i and 2k) if ignoring the amplitude modulation induced by the Coulomb attraction. This implies that in these two specific cases the Coulomb effect has tiny impact on the phase of electron wave packet and thus can be neglected in the following analysis.

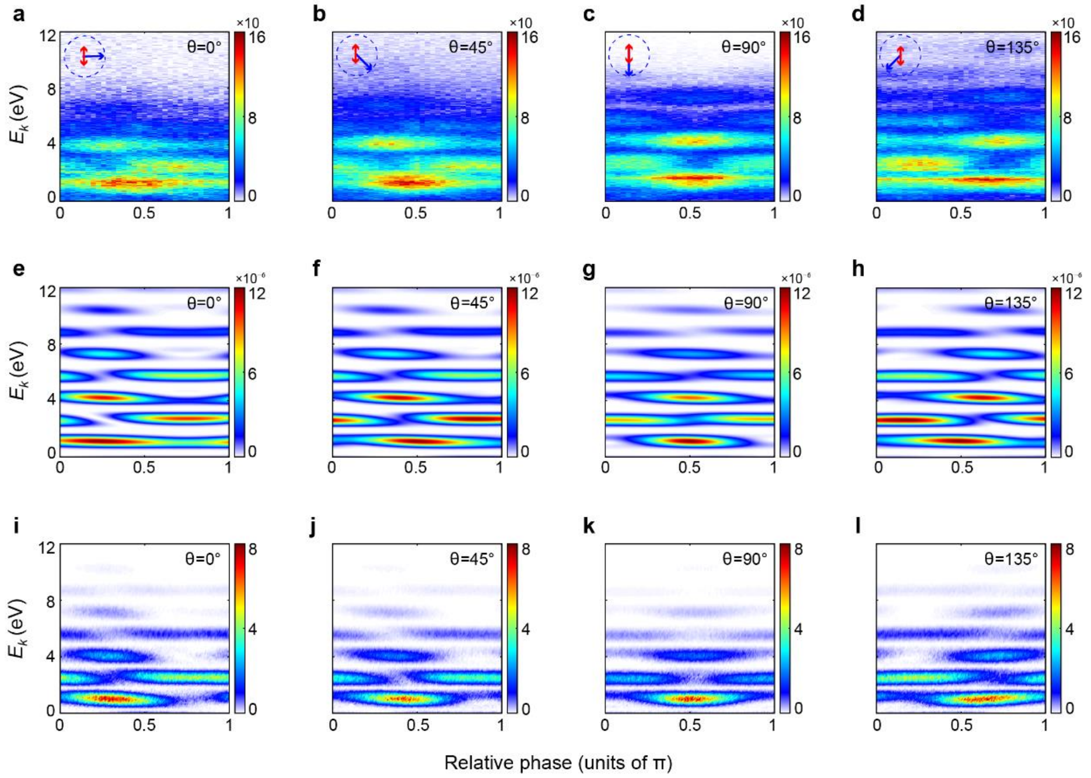

Supplementary Fig. 2| Two-color phase-resolved photoelectron energy spectra at different emission angles. a-d Experimental results. **e-h** SFA calculations. **i-l** CCSFA calculations. The polarization configurations of two-color field vectors are labeled on the top.

III. Amplitude modulation induced by the change of ρ(p) when adding a weak 800 nm field

Based on the SFA model within saddle-point approach, we can directly calculate the pre-exponential factor $\rho_s(\mathbf{p}) \sim \langle \mathbf{p}+\mathbf{A}(t_s)|\mathbf{r}\cdot\mathbf{E}(t_s)|\psi_0(\mathbf{r})\rangle$ in single- and two-color fields. Here, we label them as $\rho_s{}^0(\mathbf{p})$ and $\rho_s{}^1(\mathbf{p})$, respectively. Then, we characterize

the amplitude modulation induced by the change of pre-exponential factor using the formula of $\delta = \left| \frac{|\rho_s^{1}(\mathbf{p})| - |\rho_s^{0}(\mathbf{p})|}{|\rho_s^{0}(\mathbf{p})|} \right|$. The result of δ for parallel interaction configuration is shown in Supplementary Fig. 3a. For comparison, we also present the amplitude modulation induced by the change of imaginary part of the complex phase. Likewise, we characterize it by using $\delta = \left| \frac{e^{-\mathrm{Im}[S]} - e^{-\mathrm{Im}[S_0]}}{e^{-\mathrm{Im}[S_0]}} \right| = \left| e^{-\mathrm{Im}[\sigma]} - 1 \right|$. Here, S_0 and S represent the complex phase of electrons accumulated in single- and two-color fields. The corresponding result is shown in Supplementary Fig. 3b. One can see that the amplitude modulation induced by $\rho(\mathbf{p})$ exhibits similar phase and energy dependences as that induced by the change of imaginary part of the complex phase. However, the magnitude of amplitude modulation induced by $\rho(\mathbf{p})$ is quite smaller as compared with that induced by the change of imaginary part of complex phase. Therefore, in the following analysis we can neglect the amplitude modulation contribution induced by $\rho(\mathbf{p})$ when adding a weak 800 nm field.

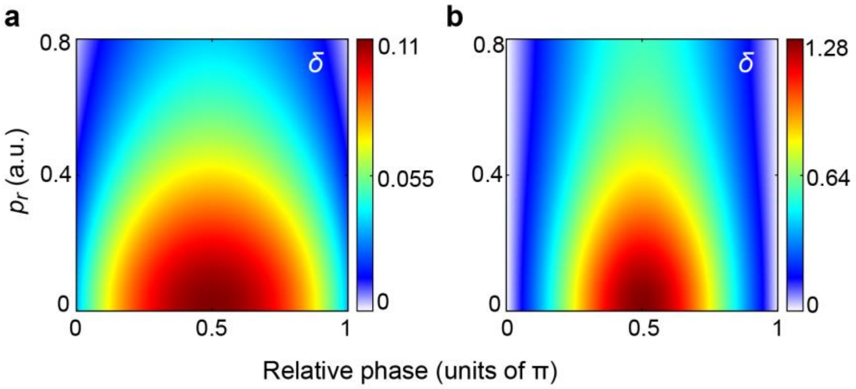

Supplementary Fig. 3| Calculated amplitude modulation δ of electron wave packet when adding a weak linearly polarized 800 nm field in parallel interaction configuration. a Amplitude modulation induced by the change of $\rho(\mathbf{p})$. **b** Amplitude modulation induced by the change of imaginary part of the complex phase.

IV. Derivation of interference formula in the employed two-color fields

As illustrated in the main text, the interference pattern in two-color fields can be well accounted for by the interference of four electron wave packets released in two

consecutive 800 nm cycles, i.e., $I(\mathbf{p},\varphi)=|\psi_1+\psi_2+\psi_3+\psi_4|^2$ as expressed in Eq. (1). As the electron wave packet can be described using $\psi=\rho(\mathbf{p})e^{iS}$, with $\rho_s(\mathbf{p})\sim\langle\mathbf{p}+\mathbf{A}(t_s)|\mathbf{r}\cdot\mathbf{E}(t_s)|\psi_0(\mathbf{r})\rangle$ denoting the pre-exponential factor and $S(\mathbf{p},t_s)=-\int_{t_s}^{\infty}[\mathbf{p}+\mathbf{A}_{400}(t)+\mathbf{A}_{800}(t,\varphi)]^2/2dt+I_pt_s$ representing the complex phase of electrons in two-color fields, we can write Eq. (1) in the main text as $I(\mathbf{p},\varphi)=|\psi_1+\psi_2+\psi_3+\psi_4|^2=|\rho_{s1}(\mathbf{p})e^{iS_1}+\rho_{s2}(\mathbf{p})e^{iS_2}+\rho_{s3}(\mathbf{p})e^{iS_3}+\rho_{s4}(\mathbf{p})e^{iS_4}|^2$. Considering the periodicity of the two-color fields, the ionization instants of the four electron wave packets satisfy $t_{s3}=t_{s1}+T_{800}$ and $t_{s4}=t_{s2}+T_{800}$. With that, the two-color synthesized fields at the ionization instants satisfy $\mathbf{E}(t_{s3})=\mathbf{E}(t_{s1})$ and $\mathbf{E}(t_{s4})=\mathbf{E}(t_{s2})$. Correspondingly, $\mathbf{A}(t_{s3})=\mathbf{A}(t_{s1})$ and $\mathbf{A}(t_{s3})=\mathbf{A}(t_{s1})$. As a result, $\rho_{3s}(\mathbf{p})=\rho_{1s}(\mathbf{p})$ and $\rho_{4s}(\mathbf{p})=\rho_{2s}(\mathbf{p})$. As for the complex phase, it is governed by the following relationship:

$$
\begin{aligned}
S_3 &= -\int_{t_{s1}+T_{800}}^{\infty}[\mathbf{p}+\mathbf{A}_{400}(t)+\mathbf{A}_{800}(t,\varphi)]^2/2dt+I_p(t_{s1}+T_{800}) \\
&= -\int_{t_{s1}}^{\infty}[\mathbf{p}+\mathbf{A}_{400}(t)+\mathbf{A}_{800}(t,\varphi)]^2/2dt+I_pt_{s1}+\int_0^{T_{800}}[\mathbf{p}+\mathbf{A}_{400}(t)+\mathbf{A}_{800}(t,\varphi)]^2/2+I_pdt \\
&= S_1+b \\
S_4 &= -\int_{t_{s2}+T_{800}}^{\infty}[\mathbf{p}+\mathbf{A}_{400}(t)+\mathbf{A}_{800}(t,\varphi)]^2/2dt+I_p(t_{s2}+T_{800}) \\
&= -\int_{t_{s2}}^{\infty}[\mathbf{p}+\mathbf{A}_{400}(t)+\mathbf{A}_{800}(t,\varphi)]^2/2dt+I_pt_{s2}+\int_0^{T_{800}}[\mathbf{p}+\mathbf{A}_{400}(t)+\mathbf{A}_{800}(t,\varphi)]^2/2+I_pdt \\
&= S_2+b
\end{aligned}
$$

. Here, $b=\int_0^{T_{800}}[\mathbf{p}+\mathbf{A}_{400}(t)+\mathbf{A}_{800}(t,\varphi)]^2/2+I_pdt$. It corresponds to the phase difference due to the time difference of traveling in the continuum and can be reduced to a constant phase with $b=(U_p^{(400)}+U_p^{(800)}+E_k+I_p)T_{800}$. Here, $E_k=|\mathbf{p}|^2/2$ denotes the photoelectron energy. U_p is the pondermotive energy, with $U_p^{(400)}=E_{400}^2/(8\omega^2)$ and $U_p^{(800)}=E_{800}^2/(4\omega^2)$. Then, the interference formula can be rewritten as:

$$
\begin{aligned}
I(\mathbf{p},\varphi) &= \left|\psi_1+\psi_2+\psi_3+\psi_4\right|^2 \\
&= \left|\rho_{s1}(\mathbf{p})e^{iS_1}+\rho_{s2}(\mathbf{p})e^{iS_2}+\rho_{s1}(\mathbf{p})e^{iS_1+ib}+\rho_{s2}(\mathbf{p})e^{iS_2+ib}\right|^2 \\
&= \left|\rho_{s1}(\mathbf{p})e^{iS_1}+\rho_{s2}(\mathbf{p})e^{iS_2}\right|^2 |1+e^{ib}|^2 \\
&= 2\left|\rho_{s1}(\mathbf{p})e^{iS_1}+\rho_{s2}(\mathbf{p})e^{iS_2}\right|^2 [1+\cos(b)]
\end{aligned}
\quad . \qquad S(1)
$$

Since 800 nm is perturbative weak, we assume it has tiny effect on the ionization instants of the electron wave packets ψ_1 and ψ_2 and the pre-exponential factor $\rho_s(\mathbf{p})$. Therefore, $t_{s2}=t_{s1}+T_{400}$, and $\rho_{s1}(\mathbf{p})\approx\rho_{s2}(\mathbf{p})$. In the following, we label $\rho_{s1}(\mathbf{p})$ and $\rho_{s2}(\mathbf{p})$ as $\rho_s(\mathbf{p})$. Correspondingly, the complex phases of the two electron wave packets, i.e., ψ_1 and ψ_2, can be expressed as

$$
\begin{aligned}
S_1 &= -\int_{t_{s1}}^{\infty}[\mathbf{p}+\mathbf{A}_{400}(t)+\mathbf{A}_{800}(t,\varphi)]^2/2dt+I_pt_{s1} \\
&= -\int_{t_{s1}}^{\infty}[\mathbf{p}+\mathbf{A}_{400}(t)]^2/2dt+I_pt_{s1}-\int_{t_{s1}}^{\infty}[\mathbf{p}+\mathbf{A}_{400}(t)]\mathbf{A}_{800}(t,\varphi)dt-\int_{t_{s1}}^{\infty}[\mathbf{A}_{800}(t,\varphi)]^2/2 \\
&= S_{10}+\sigma_1+\alpha_1 \\
S_2 &= -\int_{t_{s2}}^{\infty}[\mathbf{p}+\mathbf{A}_{400}(t)+\mathbf{A}_{800}(t,\varphi)]^2/2dt+I_pt_{s2} \\
&= -\int_{t_{s2}}^{\infty}[\mathbf{p}+\mathbf{A}_{400}(t)]^2/2dt+I_pt_{s2}-\int_{t_{s2}}^{\infty}[\mathbf{p}+\mathbf{A}_{400}(t)]\mathbf{A}_{800}(t,\varphi)dt-\int_{t_{s2}}^{\infty}[\mathbf{A}_{800}(t,\varphi)]^2/2 \\
&= S_{20}+\sigma_2+\alpha_2 \\
&= S_{10}+(E_k+U_p^{(400)}+I_P)T_{400}+\sigma_2+\alpha_1+U_p^{(800)}T_{400} \\
&= S_{10}+\sigma_2+\alpha_1+b/2
\end{aligned}
\quad . \qquad S(2)
$$

Here, $S_0=-\int_{t_s}^{\infty}[\mathbf{p}+\mathbf{A}_{400}(t)]^2/2dt+I_pt_s$ represents the phase induced solely by 400 nm circular fields. $\sigma=-\int_{t_s}^{\infty}[\mathbf{p}+\mathbf{A}_{400}(t)]\mathbf{A}_{800}(t,\varphi)dt$ is the additional phase induced by the weak 800 nm linearly polarized fields. $\alpha=-\int_{t_s}^{\infty}[\mathbf{A}_{800}(t,\varphi)]^2/2=U_{\mathrm{p}}^{(800)}t_s$ is a high-order small quantity that depends on the laser intensity of 800 nm field, therefore in the following derivation we can neglect its contribution. By substituting the phases in Eq. S(2) into Eq. S(1), one can obtain the following formula:

$$
\begin{aligned}
I(\mathbf{p},\varphi) &= \left|\psi_1+\psi_2+\psi_3+\psi_4\right|^2 \\
&= 2\left|\rho_s(\mathbf{p})e^{iS_1}+\rho_s(\mathbf{p})e^{iS_2}\right|^2 [1+\cos(b)] \\
&= 2\left|\rho_s(\mathbf{p})e^{iS_{10}+i\sigma_1}+\rho_s(\mathbf{p})e^{iS_{10}+i\sigma_2+ib/2}\right|^2 [1+\cos(b)]
\end{aligned}
\quad . \qquad S(3)
$$

Then, we use $W_0=\rho(\mathbf{p})e^{-\mathrm{Im}[S_{10}]}$ and Re[S_{10}] to denote the amplitude and phase of the

unperturbed electron wave function in 400 nm circular fields. Accordingly, the interference formula can be arranged into:

$$\begin{aligned} I(\mathbf{p},\varphi) &= |\psi_1+\psi_2+\psi_3+\psi_4|^2 \\ &= 2\left|W_0 \mathrm{e}^{i\mathrm{Re}[S_{10}]}\mathrm{e}^{i\sigma_1}+W_0 \mathrm{e}^{i\mathrm{Re}[S_{10}]}e^{i\sigma_2+ib/2}\right|^2[1+\cos(b)]. \\ &= 2W_0^{\,2}\left|\mathrm{e}^{i\sigma_1}+e^{i\sigma_2+ib/2}\right|^2[1+\cos(b)] \end{aligned} \qquad \mathrm{S(4)}$$

From this formula, one can see that the phase information (i.e., Re[S_{10}]) of the unperturbed electron wave function is lost when considering the interference effect. Further, we deduce the analytical formula of σ according to its definition for two-color fields with $\mathbf{E}(t)$=$E_{2\omega}$[cos(2ωt)$\mathbf{z}$+sin(2ωt)$\mathbf{x}$]+$E_\omega$cos(ωt+φ)$\mathbf{z}$, which is expressed as

$$\begin{aligned} \sigma(t_s) &= -\int_{t_s}^{\infty}[\mathbf{p}+\mathbf{A}_{400}(t)]\mathbf{A}_{800}(t,\varphi)dt \\ &= -\frac{p_z E_{800}}{\omega^2}\cos(\omega t_s+\varphi)+\frac{E_{400}E_{800}}{12\omega^3}\sin(3\omega t_s+\varphi)-\frac{E_{400}E_{800}}{4\omega^3}\sin(\omega t_s-\varphi) \end{aligned} . \qquad \mathrm{S(5)}$$

As t_{s2}=t_{s1}+T_{400}, we find σ_2=-σ_1. This indicates that the two electron wave packets emitted from adjacent 400 nm cycles experience opposite amplitude and phase modulations. In the following, we denote σ_1 as -σ and σ_2 as σ. Then, we rearrange the interference formula as:

$$\begin{aligned} I(\mathbf{p},\varphi) &= |\psi_1+\psi_2+\psi_3+\psi_4|^2 \\ &= 2W_0^{\,2}\left|\mathrm{e}^{-i\sigma}+e^{i\sigma+ib/2}\right|^2[1+\cos(b)] \\ &= 2W_0^{\,2}\left|\mathrm{e}^{\mathrm{Im}[\sigma]}\mathrm{e}^{-i\mathrm{Re}[\sigma]}+\mathrm{e}^{-\mathrm{Im}[\sigma]}e^{i\mathrm{Re}[\sigma]+ib/2}\right|^2[1+\cos(b)] \\ &= 2W_0^{\,2}[\mathrm{e}^{2\mathrm{Im}[\sigma]}+\mathrm{e}^{-2\mathrm{Im}[\sigma]}+2\cos(2\mathrm{Re}[\sigma]+b/2)][1+\cos(b)] \\ &= 2W_0^{\,2}[\mathrm{e}^{2\mathrm{Im}[\sigma]}+\mathrm{e}^{-2\mathrm{Im}[\sigma]}][1+\cos(b)]+4W_0^{\,2}\cos(2\mathrm{Re}[\sigma]+b/2)] \\ &\quad +2W_0^{\,2}\cos[2\mathrm{Re}[\sigma]-b/2]+2W_0^{\,2}\cos[2\mathrm{Re}[\sigma]+3b/2] \end{aligned} . \qquad \mathrm{S(6)}$$

Note that b=($U_p^{(400)}$+$U_p^{(800)}$+E_k+I_p)$T_{800,}$ we can further arrange it into the formula of b=2E_kT_{400}+2a, with a=($U_p^{(400)}$+$U_p^{(800)}$+I_p)T_{400}. Therefore, the interference formula in the two-color fields can be finally expressed as

$$\begin{aligned} I(E_k,\varphi) &= |\psi_1+\psi_2+\psi_3+\psi_4|^2 \\ &= 2W_0^2(e^{2\mathrm{Im}[\sigma]}+e^{-2\mathrm{Im}[\sigma]})[1+\cos(2E_kT_{400}+2a)] \\ &\quad +4W_0^2\cos(E_kT_{400}+2\mathrm{Re}[\sigma]+a) \\ &\quad +2W_0^2\cos(E_kT_{400}-2\mathrm{Re}[\sigma]+a) \\ &\quad +2W_0^2\cos(3E_kT_{400}+2\mathrm{Re}[\sigma]+3a) \end{aligned} . \qquad \mathrm{S(7)}$$

V. Calculated temporal shaping of electron wave functions in parallel and perpendicular configurations

Based on the derived analytical formulas as presented in Eq. (4) in Methods in the main text, we can directly calculate the two-color phase-dependent Im[σ] and Re[σ] spectra for the parallel and perpendicular configurations. The corresponding spectra are displayed in Supplementary Fig. 4. They show good agreement with the retrieved results from the SFA calculations in the main text (as shown in Fig. 3a,b and Fig. 4a,b), demonstrating the validity of the Fourier transform analysis in retrieving the amplitude and phase modulations of the electron wave function.

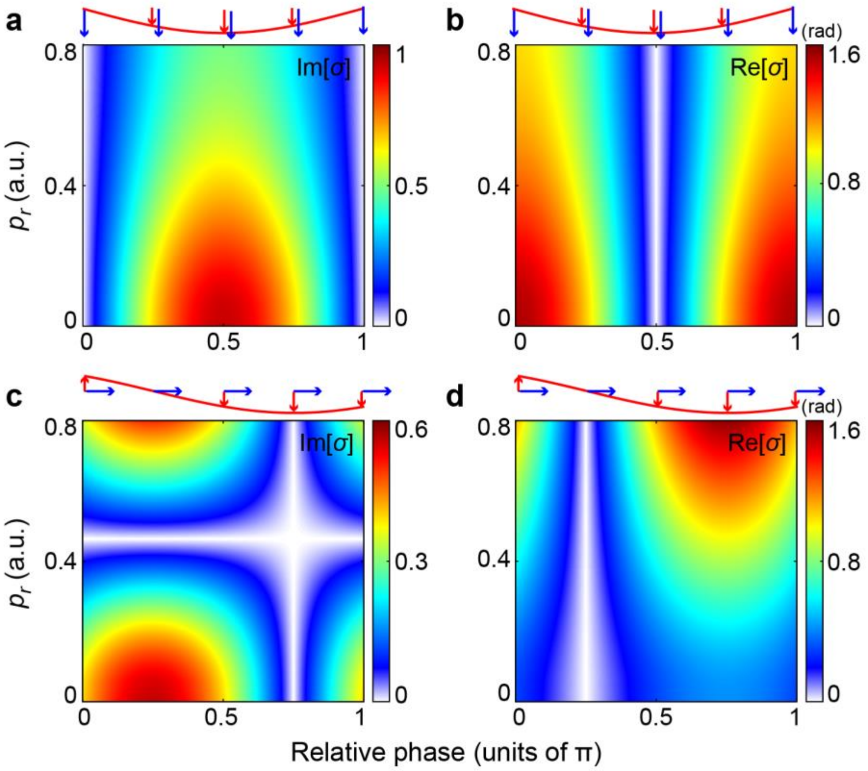

Supplementary Fig. 4| Calculated temporal shaping of electron wave function in parallel and perpendicular configurations using the SFA model. a, b Calculated amplitude modulation Im[σ] and phase modulation Re[σ] for parallel configuration. **c, d** Same as **a, b** but for perpendicular configuration. The two-color field configurations have been depicted with red and blue arrows.

VI. Calculated phase modulation of electron wave function resulting from the under-barrier motion and subsequent classical propagation

According to the definition of σ, i.e., $\sigma = -\int_{t_s}^{\infty} [\mathbf{p} + \mathbf{A}_{400}(t)] \cdot \mathbf{A}_{800}(t,\varphi) dt$, one could see the phase modulation Re[σ] actually results from the entire ionization process. Within the saddle-point approach, we can divide σ into two parts, i.e., $\sigma = \sigma_1 + \sigma_2$, with $\sigma_1 = -\int_{t_s}^{t_r} [\mathbf{p} + \mathbf{A}_{400}(t)] \cdot \mathbf{A}_{800}(t,\varphi) dt$ representing the additional phase accumulated during the under-barrier motion and $\sigma_2 = -\int_{t_r}^{\infty} [\mathbf{p} + \mathbf{A}_{400}(t)] \cdot \mathbf{A}_{800}(t,\varphi) dt$ denoting the additional phase accumulated in the classical propagation. Here, t_r is the real part of t_s with $t_s = t_r + it_i$. The calculated two-color phase-resolved phase modulations in the under-barrier motion and subsequent classical propagation, i.e., Re[σ_1] and Re[σ_2], are shown in Supplementary Fig. 5 for parallel and perpendicular interaction configurations. Clearly, one could see that, in parallel interaction configuration, the phase modulation resulting from the under-barrier dynamics (Supplementary Fig. 5a) is much larger than that in the classical propagation (Supplementary Fig. 5b). This indicates in parallel interaction configuration, the retrieved Re[σ] spectra as shown in Fig. 3b and 3d in the main text actually reflect the main features of the real phase modulation accumulated during under-barrier motion. While for the perpendicular interaction configuration, the situation reverses. It is shown that the phase modulation accumulated in the classical propagation (Supplementary Fig. 5d) is larger than that resulting from the under-barrier dynamics (Supplementary Fig. 5c). This means the retrieved phase modulation Re[σ] (as shown in Fig. 4b and 4d in the main text) mainly comes from the classical propagation.

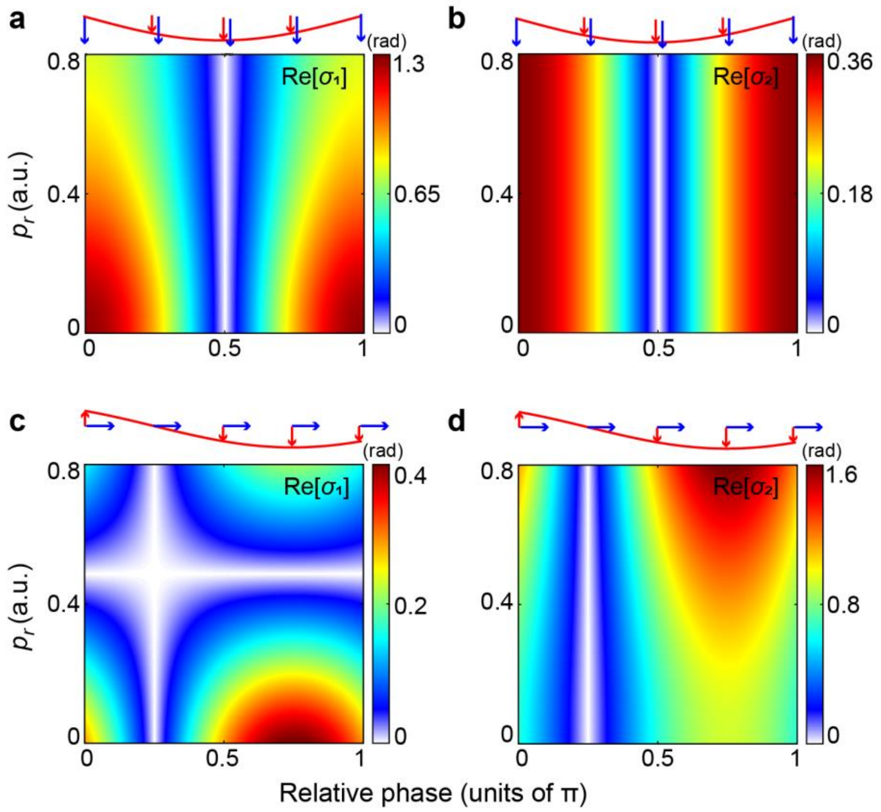

Supplementary Fig. 5| Calculated temporal evolution of the phase modulations resulting from the under-barrier dynamics and the subsequent classical propagation for parallel and perpendicular interaction configurations. a, b Calculated two-color phase (time)-dependent Re[σ_1] and Re[σ_2] for parallel configuration. **c, d** Same as **a, b** but for perpendicular configuration. The two-color field configurations have been depicted with red and blue arrows.

VII. Calculated angle-resolved photoelectron momentum spectra by SFA model and CCSFA model

In order to identify the Coulomb effect, we have calculated the angle-resolved photoelectron momentum spectra in two-color fields with φ=0 using SFA model and CCSFA model. The corresponding results are shown in Supplementary Fig. 6. Here, to clearly visualize the deflection of electron wave packet, we have neglected the interference effect. It is clearly shown that, influenced by the Coulomb potential, the radial momentum (energy) of the electron wave packet decreases, and at the same time the electron wave packet is deflected by a specific angle. By inspecting the most

probable emission angle at which the photoelectron yield reaches the maximum, one can obtain the deflection angle. Then, by rotating the experimental spectrum with this angle, we can roughly eliminate the Coulomb effect.

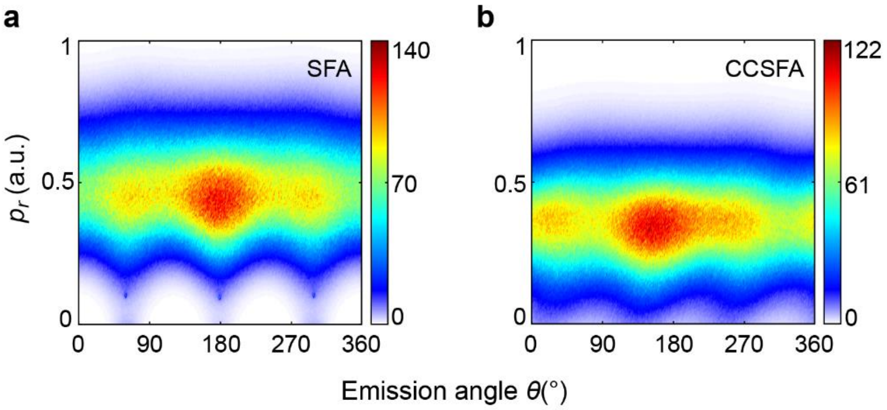

Supplementary Fig. 6| Calculated angle-resolved photoelectron momentum spectra for two-color fields with φ=0. a SFA calculation. **b** CCSFA calculation.
